# Survival analysis to measure turnover of the medical education workforce in Ethiopia

**DOI:** 10.1186/s12960-017-0197-0

**Published:** 2017-03-14

**Authors:** Tsion Assefa, Damen Haile Mariam, Wubegzier Mekonnen, Miliard Derbew

**Affiliations:** 1 School of Public Health, Addis Ababa University, PO Box 9086, Addis Ababa, Ethiopia; 2 School of Medicine, Addis Ababa University, PO Box 9086, Addis Ababa, Ethiopia

**Keywords:** Ethiopia, Health workforce, Medical education workforce, Specialist, Faculty physician/physician, Physician turnover/retention, Physician migration, Survival analysis

## Abstract

**Background:**

Until recently, there were only a few medical schools in Ethiopia. However, currently, in response to the apparent shortage in physician workforce, the country has made huge progress with respect to the expansion of medical schools, by adopting the so-called flooding strategy. Nevertheless, the effectiveness of the intended strategy also relies on physician accessibility and turnover. Therefore, the aim of this study was to examine the distribution of physicians in the medical schools of Ethiopia and to quantify the magnitude and identify factors associated with physician turnover.

**Methods:**

This organizational faculty physician workforce survey was conducted in seven government-owned medical schools in Ethiopia. Longitudinal medical workforce data set of about 6 years (between September 2009 and June 2015) were retrospectively collected from each of the medical schools. The observation time begins with the date of employment (time zero) and ends at the date on which the physician leaves the appointment/or the data collection date. Kaplan-Meier survival method was used to describe the duration of stay of physicians in the academic health care settings. A Cox proportional hazards (CPH) model was fitted to identify the risk factors for physician turnover.

**Results:**

In this study, a total of 1258 faculty physicians were observed in seven medical schools which resulted in 6670.5 physician-years. Of the total, there were 198 (15.7%) turnover events and the remaining 1060 (84.3%) were censored. The average turnover rate is about 29.7 per 1000 physician-years of observations.

Multivariate modeling revealed no statistical significant difference in the rate of turnover between males and females (adjusted hazard ratio (AHR), 1.12; 95%CI, 0.71, 1.80). However, a lower rate of physician turnover was observed among those who were born before 1975 (AHR, 0.37; 95%CI, 0.20, 0.69) compared with those who were born after 1985. Physicians with the academic rank of associate professor and above had a lower (AHR, 0.25; 95%CI, 0.11, 0.60) rate of turnover in comparison to lecturers. In addition, physicians working in Jimma University had 1.66 times higher rate of turnover compared with those working in Addis Ababa University. However, the model showed a significantly lower rate of turnover in Mekelle (AHR, 0.16; 95%CI, 0.06, 0.41) and University of Gondar (AHR, 0.46; 95%CI, 0.25, 0.84) compared with that of Addis Ababa. Physician turnover in the remaining medical schools (Bahir Dar, Haromaya, and Hawassa) did not show a statistically significant difference with Addis Ababa University (*P* > 0.05).

**Conclusions:**

This study revealed a strong association between physician turnover with age, academic rank, and workplace. Therefore, the findings of the study have important implications in that attention needs to be given for the needs of faculty physicians and for improving the work environment in order to achieve a high level of retention.

## Background

Medical education has a relatively short history in Ethiopia. This is reflected not only by the small number of medical schools, only three for several years, but also by the small number of medical doctors which have been produced. Since the establishment of the first three medical schools (Faculty of Medicine, Addis Ababa University (AAU) in 1964, Gondar Public Health College in 1978, and Jimma Medical School in 1983) to 2006, the medical schools were able to produce only less than four thousand medical doctors [1–3]. Besides, attractive overseas remuneration, non-governmental organizations (NGOs), or the private sector pulled the majority of the physician workforce out of the public health sector [2, 4]. This incidence, together with low production, left the country in severe physician workforce shortages and crisis.

However, following the apparent shortages and crisis, the country began to work on health workforce retention and production. Salary increment, incentives according to geographical location, and further training opportunities have been set as retention strategies [5]. Moreover, massive increase has been made in medical education expansion which started with the establishment of the two medical schools in Mekelle (MU) and Hawassa (HwU) Universities in 2003 by involving the private sector. The number of medical schools grew from three to more than 25 [6] using the so-called flooding strategy [7].

By its very nature, clinical service provision and medical education are human resource intensive. This claims a human resource development strategy that can possibly fit to the context, in a way that balances the supply and demand that means, taking into account the economic and health system demand to accommodate large number of graduates [8]. And on the supply side, medical training demands good composition of medical instructors and their relative stay in the medical schools along with other necessary resources [9, 10].

This research intends to add to the existing body of little knowledge in resource-limited settings. For instance, in the Ethiopian context, in organizationally embedded teaching hospitals, medical teachers are professional physicians (the medical education workforce have dual roles—both as instructors of the medical students and clinical service providers). Thus, there should be many physicians with various specialty and sub-specialty trainings coupled with rich experience in their field of specialty in these teaching hospitals. We need, therefore, to examine the availability (in required number, qualification, and composition) of physicians in the medical schools of Ethiopia and identify factors associated with their turnover which warrants this study.

Balancing health workforce distribution and retention strategies through evidence-based intervention in the academic health care setting mostly depends upon different factors: firstly, on the availability of continuous data and the practice of analyzing the data to understand the situation; secondly, on the experience of transforming such data into valuable information for informed decision making [11] which is very important for making policy decisions on medical education expansion and to strengthening the existing medical schools; and finally, on the system’s commitment in using evidence-based interventions to improve the situation together with the knowledge of their effectiveness. Many developing countries including Ethiopia, however, lack these important interlinked components of health human resources management.

In Ethiopia, the medical education expansion to address the severe workforce shortage would have been complete if adequate evidence were taken into consideration, particularly on the medical education workforce composition and the effect of the massive admissions to medical schools on physician workforce performance (as instructors of medical students and clinical care providers). Such evidence would also inform the effectiveness of human resource retention strategies in reducing the turnover. Therefore, this study tries to show the distribution of medical education workforce by their different characteristics and their turnover and retention.

## Methods

### Settings

This study involved seven government-owned medical schools in Ethiopia. Each medical school has organizationally embedded teaching hospitals (in the country’s health care tier system, teaching hospital provides the highest/tertiary level health care; also called specialized hospital) with the exception of Bahir Dar University (BDU). For instance, the Faculty of Medicine, AAU, which is the biggest medical school in the country owned Tikur Anbessa Specialized Hospital which is the biggest referral hospital in the country. The School of Medicine, University of Gondar (UOG), is located in Amhara Region, Northwest Ethiopia. Its teaching hospital serves as one of the referral hospitals in the region. Similarly, the School of Medicine, BDU, is located in Bahir Dar city, the capital of Amhara Region. At the time of this study, this medical school did not have its own teaching hospital though it has been using Felege Hiwot Referral Hospital, owned by the regional health bureau.

**Fig. 1:**
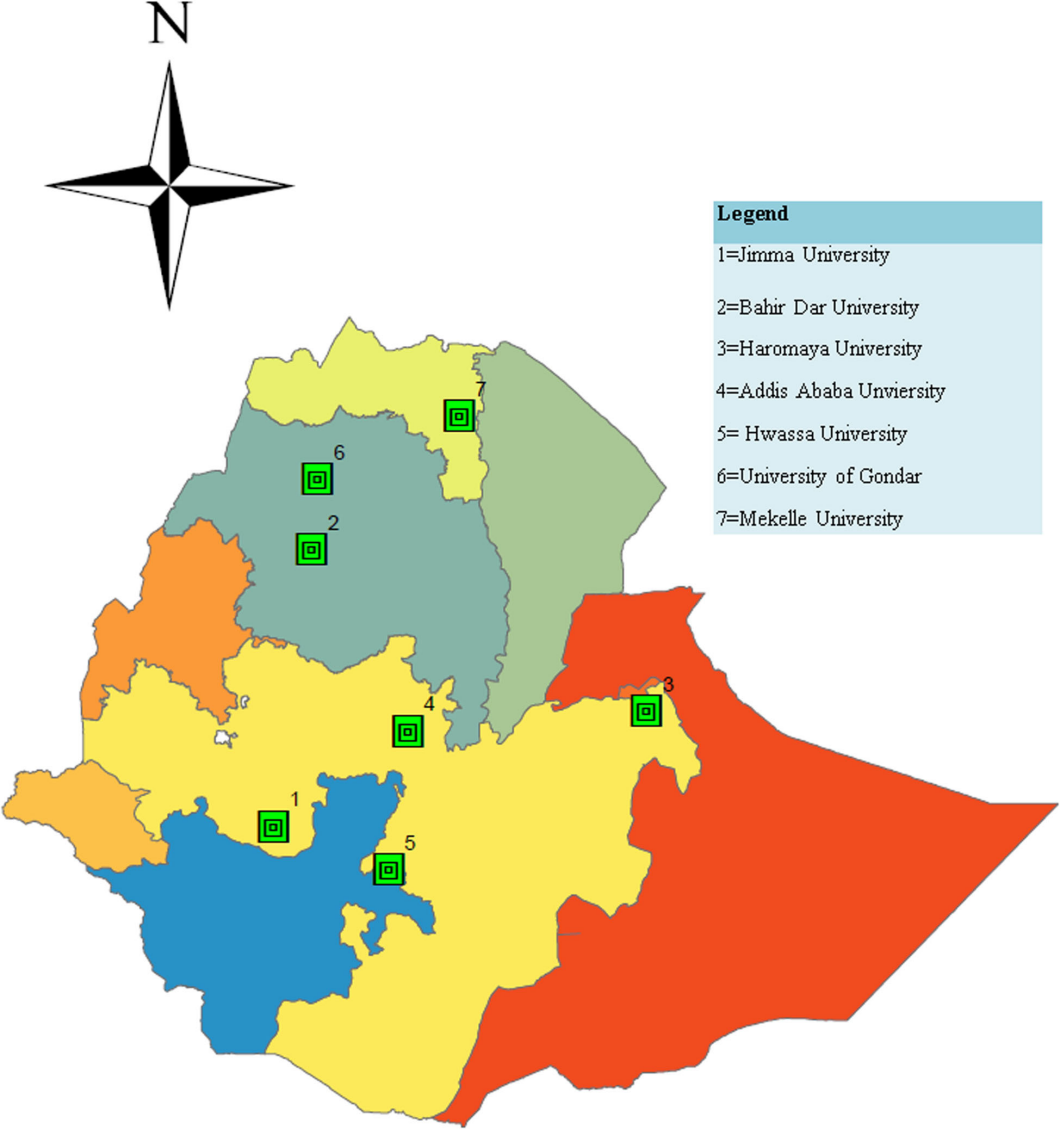
Location of the medical schools involved in the study

Similarly, the School of Medicine, MU, is located in Mekelle city, the capital of Tigray Region. Its teaching hospital is called Ayder Referral Hospital, the biggest referral hospital in the region. The School of Medicine, Jimma University (JU), is located in Jimma town, Southwest Ethiopia. Its teaching hospital is also the biggest referral hospital in Southwest Ethiopia. The School of Medicine, HwU, is located in Hawassa city, the capital of Southern Nations, Nationalities, and Peoples Region (SNNPR) of Ethiopia. The teaching hospital serves as a referral hospital for the community residing in the region and around. Likewise, the School of Medicine, Haromaya University (HU), is located in the old Harar city, Harari Region. And its teaching hospital, Hiwot Fana Referral, provides clinical service for the population residing in the eastern provinces of the country (Fig. 1). At the time of the study, each medical school had an average of 578 pre-clinical and 589 clinical year students. In addition, the proportion of foreign and contract staffs were varied across the medical schools and estimated from less than 1 to 13.5% of the workforce (Table 1).

**Table 1:** Background information of the medical schools with their respective teaching hospitals involved in the study

S.No	Name of the university	Year established	Pre-clinical and clinical year students (2014/2015)		Name of teaching hospital	Teaching staff			
						Expatriate	Contract	Non-academic	Academic
1.	Addis Ababa	1964	926	776	Tikur Anbessa Specialized Hospital	10	–	4	320
2.	Gondar	1978	564	616	Gondar Referral Hospital	1	3	14	164
3.	Jimma	1983	631	568	Jimma University Specialized Hospital	13	1	21	120
4.	Mekelle	2003	489	637	Ayder Referral Hospital	5	–	2	160
5.	Hawassa	2003	503	477	Hawassa Referral Hospital	2	4	2	116
6.	Haromaya	2007	550	580	Hiwot Fana Referral Hospital	–	–	6	65
7.	Bahir Dar	2007	386	470	Felege Hiwot Referral Hospital	2	18	NA	115

Sources: *FMOE*, annual educational abstract, 2012/2013 and the medical schools

− information not accessed, *NA* not applicable

### Study design

This organizational medical education workforce survey was conducted between February and June, 2015. Despite the importance and global recognition of the Human Resource Information System (HRIS) [11], Ethiopia does not yet have a well-organized Human Resources for Health (HRH) database system. Thus, this particular study retrospectively organized longitudinal data by collecting the available records from the human resource departments of the study medical schools. In measuring the medical education workforce turnover and retention, we employed the concepts in survival analysis [12]. In this study, the event of interest was “turnover of physicians from the medical schools in which they had been working between September 2009 and June 2015.” The observation time begins with the date of employment (time zero) and ends at the date in which the health worker leaves the appointment/or were observed during data collection.

### Data

Currently, the health workforce data are at the stage of transition from rudimentary paper-based system to capturing with excel spreadsheet using computer so that the data were not easily amenable for use in health workforce planning and undertaking research. For this study, individual practitioner level data were collected from the human resource units/departments of the study medical schools. The data have two main components: inflows and outflows. The inflow data are recorded to each physician when the physician takes up the position in the medical school, and the outflow data are recorded at the time when the physician leaves the position.

The data have also milestone characteristics including faculty physicians training opportunities, distribution, and turnover. Moreover, socio-demographic variables such as date of birth, gender, and date of appointment were commonly available including the field of specialties. However, some demographic variables such as marital status, ethnicity, and the names of institutions at which they had their residency training and fellowship were not completely recorded.

### Operational definitions

- *Medical education workforce*: refers to physicians (medical doctors) who teach medical students and provide direct patient/clinical care to patients/clients in the affiliated teaching hospitals.
- *Actively working faculty physicians/medical education workforce*: those physicians who were currently (during the data collection) working in the medical schools as medical instructors and clinical service providers.
- *General practitioner* (*GP*): refers to a medical doctor who qualified as a general practitioner or a physician who does not specialize in one particular area of medicine.
- *Specialist*: one who completed medical education in a certain area of medical specialty (such as internal medicine, surgery, pediatrics, gynecology, and obstetrics) after being trained/having served as a GP.
- *Sub-specialist*: one who received training in a certain sub-specialty area after being trained/having served as a GP and/or specialists.
- *Turnover*: a transition made (in the form of official resignation, transfer by a physician after taking up appointment within the time of interest).
  - *Officially left*: refers to those who left a certain medical school by getting permission from authorities and whose whereabouts and reasons for leaving are known; the one who took a release paper officially.
  - *Runaway*: refers to those faculty physicians who left from the medical schools without letting the office know their whereabouts while they leave.
  - *Retired*: refers those who depart from the medical school due to retirement.
  - *Transferred*: indicates movement of physicians within the public health sector, could be to the academic or non-academic health care settings.
  - *Died*: refers to the departure of a physician from the medical school because of death.
  - *Unrecognized*: refers to a turnover not recognized by the HR office before the date of data collection.
  - *Unspecified*: refers to lack of reliable information in the HR department to label the event under any of the above categories.
- Duration of stay
  - For actively working medical education workforce, duration of stay in the assigned place is calculated by subtracting the date of data collection from the date of employment.
  - For those who left their place of appointment, duration of stay is calculated using the date of departure.
  - For those with unknown dates of attrition, service year was subtracted from the date of data collection.

### Data analysis

After completing the data processing and edition in excel spreadsheet, data were imported into Stata Version 13 [13] using Stat transfer version9. Descriptive statistics were used to describe the characteristics of actively working medical education workforce/trainer physicians, the inflows and outflows of physicians, to compare educational levels, and to illustrate differences across medical schools.

### Survival analysis

Survival analysis is convenient for studies with time to event data. If study participants are unable to get enrolled at the start of observation time and did not leave before the end of the observation, retire, or die during the follow-up, then they will be censored [14]. “Retention/stay in” is the duration in which faculty physicians were working in their place of appointment, while “turnover/migration” refers the time at which they left their place of appointment.

*Event* implies “the turnover/migration of a physician from the academic health care setting.” Here in this survey, there may be repeated transitions and taking up of positions in different medical schools which were considered as an episode of independent observation. The date of employment is considered as the beginning time of the observation with time = 0 and ends at the date on which the physician leaves the appointment (September 10, 2009, and June 30, 2015)/or the date of data collection. The ending time was limited for two possible reasons: the first reason was to examine the recent physician turnover experience from the medical schools and the second reason was the availability and completeness of the turnover data.

The Kaplan-Meier survival curve was used to describe the survival/length of stay of physicians in the medical schools by their academic ranks. In this study, Cox’s proportional hazards (CPH) model was employed because the model is semi-parametric that allows for no assumptions to be made about the baseline distribution; and its flexibility to handle censoring of the survival time is due to its use of the partial likelihood function. This was important to our study in that any temporal biases due to differences in the date of employment (delayed entry to the system) for different physicians over the period; also, it allows investigating the effect of covariates by controlling other confounding variables [14]. The Cox proportional hazard model was run to identify the risk factors for physicians leaving their appointment. In fitting the model, first we set the data as panel which is a requirement in survival analysis. For categorical variables, dummy variables were created which were used to measure the association using the reference category of choice (for example, gender was coded as males = 1 (reference), females = 2); academic rank was categorized into three (lecturers = 1 (reference), assistant professors = 2, and associate professors and above = 3); date of birth is also categorized into three (born after 1985 = 1 (reference), between 1975 and 1985 = 2, and before 1975 = 3); and for medical schools, Addis Ababa University is chosen as a reference and the other medical schools are given subsequent numbers.

The Cox proportional hazard model was run using an enter method model building approach, hazard ratios (with 95%CI, and *P* value <0.05 as cut-off points) were used to explain the observed significant differences, and Breslow’s approximation was used to handle ties. The proportional hazard assumption was checked, and then posttest of proportional hazards assumption was run. The *P* value for global test equals to 0.466, indicating validity of proportional hazard assumptions. However, two variables, educational level and service year, were excluded from the model because they were found collinear with the academic rank.

## Results

During the observation period of the study, between September 2009 and June 2015, there were a total of 1258 faculty physicians in seven medical schools. A total of 6670.5 physician-years of observation were analyzed for the study period, 198 (15.7%) observations were completed, and the remaining 1060 (84.3%) were censored. The average turnover rate is about 29.7 per 1000 physician-years of observations.

### Characteristics of actively working faculty physician workforce

Of the total 1060 observations (actively working faculty physicians during the study), the majority 877 (82.8%) were males and the remaining 182 (17.2%) were female physicians. Younger physicians who were born after 1985 accounted for nearly half of the faculty physician workforce 501 (47.3%), while those born prior to 1975 were only less than 20%, 207 (19.5%).

In terms of work experience, 461 (43.5%) had worked for less than 3 years, while 335 (31.6%) had served the medical schools between 3 and 6 years, whereas only very few proportion 74 (6.9%) had worked for 15 or more years.

Regarding the educational levels, GPs constituted 61.7% of the actively working academic physician workforce, while specialists/sub-specialists constituted only 38.3%. The study has also revealed that lecturers/junior faculties constituted the large majority, 61.4%, while associate and full professors represent only 4.2% and less than 1% of the teaching physician workforce, respectively (Table 2).

**Table 2:** Characteristics of actively (during the study) working faculty physicians in the medical schools of Ethiopia, Feb–June 2015

Variable	Characteristics	Frequency	Percent
Gender	Male	877	82.8
	Female	182	17.2
Date of birth	Prior to 1975	207	19.5
	1975–1985	338	31.9
	After 1985	501	47.3
Service years	<3	461	43.5
	3–6	335	31.6
	7–10	152	14.3
	11–14	32	3.0
	>=15	74	6.9
Educational level	GPs	653	61.7
	Specialist/sub-specialist	407	38.3
Academic rank	Lecturers	651	61.4
	Assistant professors	354	33.4
	Associate professors	45	4.2
	Full professors	10	0.9
	Total of each variable	1 060	100.0

The number of physicians varied across the medical schools involved in the study. AAU had 320 (30.2%) of the total medical education workforce followed by the University of Gondar 164 (15.5%) and Mekelle 160 (15.1%). In terms of level of specialty, 59.2% of the teaching physician workforce at AAU were specialists/sub-specialists, 30.7% were residents, and only 10% were general practitioners. On the other hand, the proportions of specialists/sub-specialists in the other universities ranged from 49.2% in Jimma to below 20% in Haromaya and Bahir Dar. Similarly, significant proportions of the medical education workforce were also continuing their education (either in the residency or fellowship programs) (Table 3).

**Table 3:** Distribution of actively working faculty physicians in three main categories: GPs, residents, specialists/sub-specialists in Ethiopia, Feb–June 2015

Medical school	GPs		Residents/fellows		Specialists/sub-specialists		Total	
	Frequency	Percent	Frequency	Percent	Frequency	Percent	Frequency	Percent
AAU	32	10.0	98	30.7	190	59.2	320	30.2
UOG	34	20.7	87	53.1	43	26.2	164	15.5
MU	23	14.4	85	53.1	52	32.5	160	15.1
JU	15	12.5	46	38.3	59	49.2	120	11.3
HWU	24	20.7	49	42.2	43	37.1	116	11.0
BDU	64	55.6	41	35.6	10	8.7	115	10.9
HU	26	40	29	44.6	10	15.4	65	6.1
Total	218	100	435	100	407	100	1 060	100

*Abbreviations*: *AAU* Addis Ababa University, *BDU* Bahir Dar University, *HU* Haromaya University, *HwU* Hawassa University, *JU* Jimma University, *MU* Mekelle University, *UOG* University of Gondar

**Fig. 2:**
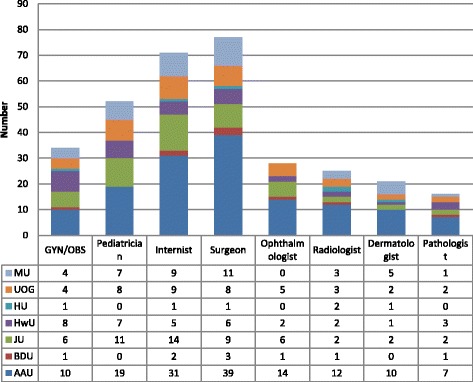
Distribution of faculty physicians by different areas of specialty category across the medical schools between Feb–June 2015

The study also examined the distribution of medical education workforce across the common clinical specialty areas (internal medicine, surgery, obstetrics and gynecology, and pediatrics) (Fig. 2). At AAU, the highest number of specialists/sub-specialists was observed in the area of surgery (39) and the least were in pathology (7). In the area of obstetrics and gynecology, the distribution ranges from 10 at AAU to only one at Bahir Dar and Haromaya. The two latter medical schools did not also have a specialist in pediatrics and child health at the time of the study. Moreover, Haromaya medical school did not have specialists of pathology and ophthalmology. Mekelle medical school did not also have a specialist in the field of ophthalmology at the time of the study.

### Characteristics of faculty physician turnover

Table 4 showed the overall turnover rates among the studied medical schools. It indicates that the turnover rate is much higher among males (20.1%) than females (11.5%). On average, the turnover rate among physicians born during 1975–1985 was 29.3%, while the same rate was only 7% for those physicians whose birthdates were after 1985.

**Table 4:** Faculty physician turnover rates between Sep 2009 and June 2015

Variable	Characteristics	Frequency	Percent
Gender	Male	177	20.1
	Female	21	11.53
Date of birth	Prior to 1975	54	26.08
	1975–1985	99	29.28
	After 1985	35	6.98
Service years	<3	65	14.1
	3–6	84	25.1
	7–10	22	14.5
	11–14	9	28.1
	>=15	17	23.0
Educational level	GPs	55	8.4
	Specialists/sub-specialists	143	35.1
Academic rank	Lecturers	55	8.4
	Assistant professors	134	37.9
	Associate professors	7	15.6
	Full professors	2	20
Medical schools	AAU	66	20.6
	JU	52	43.3
	HwU	22	19.0
	HU	18	27.7
	UOG	17	10.4
	BDU	17	14.8
	MU	6	3.8
Turnover categories	Officially left	92	46.2
	Runaway	82	41.7
	Unspecified	7	3.5
	Retired	6	3.0
	Transferred	6	3.0
	Unrecognized	4	2.1
	Died	1	0.5

On the other hand, the rate of turnover did not also show uniformity across service years. It is much higher among those who worked as faculty physician for the duration of 3–6 and 11–14 years, which is 25.1 and 28.1%, respectively, whereas the turnover rate among physicians who served their respective medical schools for less than 3 and between 7 and 10 years is nearly identical, 14.1 and 14.5%, respectively.

The turnover rate was much higher among specialists/sub-specialists than GPs. On average, the turnover rate among specialists/sub-specialists was 35.1% whereas among GPs, it was only 8.4%. Within academic ranks, more than twofold turnover rate was observed among assistant professors, 37.9%, compared with associate professors and full professors, 16.4%.

In addition, the turnover rate also varied among study medical schools. The highest turnover rate was observed in Jimma medical school 43.3%, followed by Haromaya 27.7%, whereas much lower turnover rate was observed in Mekelle and the University of Gondar medical schools, which was only 3.8 and 10.4%, respectively. In addition, the study also examined the procedures used for the move out from the study medical schools. Some obtained official permission while others did it without permission. Official permission through legal release was secured among 46.2% of the out migrant physicians whereas 41.7% of them did it without even informing their respective medical schools. And only very few proportion departed through inevitable events, such as retirement (3%) and death (0.5%) (Table 4).

### Findings from the survival analysis

**Fig. 3:**
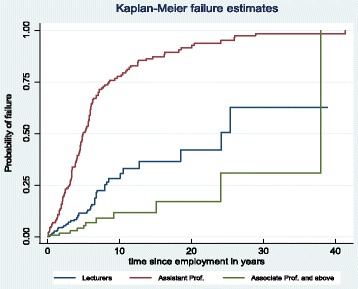
Medical education workforce by academic rank adjusted for educational level

Figure 3 illustrates a turnover pattern for academic physicians working as lecturers, assistant professors, and associate and full professors after adjusting for educational levels. The log-rank test is statistically significant (*X* ^2^ = 24; *P* < 0.001), that means the turnover rate varies between the academic ranks. The turnover rate was higher among the middle rank (assistant professors), followed by the lower rank (lecturers) and the highest academic rank associate and full professors. The sharp increase in the turnover pattern among assistant professors was noticeable in the first 10 years of service. In fact, the attrition rate has been fast in the early years of service for all academic ranks portrayed in Fig. 3. The turnover curve was steadily increasing for physicians working for 10 to 27 years and leveled for those working between 27 and 38 years indicating a very low turnover rate among this group. In the end, the line goes up vertically which indicate a natural separation for those who reached to the age of retirement.

Univariate analyses revealed statistically significant (*p* < 0.05) differences in the turnover rates of faculty physicians by their gender, birth year, educational level, academic rank, and medical school groups. When multivariate Cox’s proportional hazard model was fitted to identify factors associated with physician turnover after controlling other variables, no statistical significant difference was observed in the rate of turnover between males and females (AHR, 1.12; 95%CI, 0.71, 1.80). However, the model revealed that those who were born prior to 1975 had a 63% lower rate of turnover (AHR, 0.37; 95%CI, 0.20, 0.69) compared with physicians born after 1985. No statistical significant difference in the rate of physician turnover was revealed between those born during 1975–1985 and the reference group, who were born after 1985 (*P* > 0.05). Physicians with the academic rank of associate professors and above were also associated with a 75% lower (AHR, 0.25; 95%CI, 0.11, 0.60) rate of turnover compared with lecturers but not for assistant professors compared with the reference group, lecturers (*P* > 0.05).

With regard to differences in the risk of turnover across medical schools, physicians working in Jimma had 1.66 times increased rate of turnover compared with the rate of those at AAU (AHR, 1.66; 95%CI, 1.08, 2.55). On the other hand, physicians in Mekelle had an 84% decreased risk of turnover and those at the University of Gondar had a 54% decreased risk of turnover compared with the reference group, AAU with the following values (AHR, 0.16; 95%CI, 0.06, 0.41) and (AHR, 0.46; 95%CI, 0.25, 0.84), respectively. However, in the risk of physician turnover in the remaining medical schools (Bahir Dar, Haromaya, and Hawassa) was not statistically significantly different compared with the risk of turnover among physicians working in the Medical Faculty of Addis Ababa University (*P* > 0.05) (Table 5).

**Table 5:** Cox proportional hazards model: risk factors for faculty physician turnover, Sep 2009 and June 2015

Variables	Category	Adjusted hazard ratio (AHR)	SE	*P* value	LL 95%CI	UL 95%CI
Gender	Males (Ref.)					
	Females	1.12	0.27	0.60	0.71	1.80
Date of birth	After 1885 (Ref.)					
	1975–1985	0.91	0.23	0.73	0.56	1.49
	Prior 1975	0.37	0.11	0.002*	0.20	0.69
Academic rank	Lecturers (Ref.)					
	Assistant professors	1.03	0.24	0.89	0.66	1.61
	Associate professors and above	0.25	0.11	0.002*	0.11	0.60
Medical schools	AAU (Ref.)					
	BDU	1.12	0.34	0.70	0.62	2.04
	UOG	0.46	0.14	0.01*	0.25	0.84
	HU	1.25	0.37	0.44	0.70	2.24
	HwU	0.84	0.23	0.54	0.49	1.45
	JU	1.66	0.36	0.02*	1.08	2.55
	MU	0.16	0.07	0.00**	0.06	0.41

*n* = 198

*Ref* reference group, *SE* standard error, *CI* confidence interval, *LL* lower limit, *UL* upper limit

*p < 0.05; **p < 0.001

## Discussion

Using a longitudinal retrospective data, this study showed the characteristics of medical education workforce in Ethiopia. Our results confirm that the medical education workforce is composed of predominantly males, young, and less experienced faculty physicians. The medical education workforce composition and turnover significantly varied across the studied medical schools, even among the long-standing medical schools (the turnover rate has been average in Addis Ababa, higher at JU, and lower in UOG and Mekelle). This might have an important implication for managing HRH turnovers in the teaching health care settings, in creating uniform and conducive work environment across the medical schools, and for improved patient outcomes at teaching hospitals and for the better attainment of medical students. And also, this is valuable evidence for making informed decision in the expansion of medical education.

In the study, general practitioners and residents share substantial proportion of the medical education workforce of the country. Nevertheless, the turnover rate was seen to be significantly lower among younger and less experienced physicians. This may indicate the presence of improved supply in physician workforce and/or use of compulsory service schemes delayed the turnover of younger physicians from the public sector [15].

On the other hand, even though the proportion of physicians who reached to the highest academic ranks (associate and full professors) were very low, the rate of turnover among them was comparatively lower. This finding may suggest low incentive packages as potential sources of inducing turnover as reported elsewhere [16–18]. In addition, it is also possible that senior physicians in teaching and research might be relatively more satisfied in their carriers to stay longer in their positions, indicating the needs for rewarding excellence and professional standards for retaining the physician workforce [10, 19].

The present study has also identified shortages and lack of diversity in medical education workforce. The shortage of specialists in the areas of pediatrics and child health and obstetrics and gynecology are specially concerning since these areas are the most demanding and critical to address the maternal and child health needs of the country [20]. Furthermore, there are also evidence in the literature regarding the importance of diversity in the medical workforce for improved patient outcomes and for better educational attainment of medical students [19]. These findings also imply that instead of mere expansion of medical schools without having appropriate medical teaching workforce, it is important to fulfill basic requirements and to address the critical incentive needs of the teaching workforce [9].

The results of the Kaplan-Meier survival curve showed statistically significant difference in the rate of turnover between the academic ranks. In addition, the findings from the Cox proportional hazard model showed lower risk of turnover among older and higher academic ranks, indicating the need for focusing in research-oriented retention strategies and for working more on retaining younger physicians because they are the successors of the retiring seniors. Furthermore, the Cox proportional hazard model shows variations in the rate of turnover among the schools included in the study. This might be a reflection of the different working environments within the schools, despite the fact that all of them are owned and administered by the same government ministry (an issue of serious concern). In particular, high rates of turnover within the longstanding medical schools might reflect the presence of pushing factors in the work environment [20, 21], in addition to issues of management that relate to the retention of the medical education workforce [22]. The descriptive findings might also support such justifications since about 41.7% of the physicians left their appointments without notifying their reasons [15].

Overall, to be effective in human resource development, two major health system efforts, physician recruitment and retention, should go side by side [23], in addition to the need for reducing the pull factors by improving the supply and push factors by strengthening health care system which is also necessary [24–26].

### Limitations

This study has two potential limitations: it did not address the performance and was not able to capture the whereabouts of those who left their appointments and the medical schools did not also have well-organized HRIS database.

## Conclusions

The studied medical schools provide not only medical education to the medical students but also clinical care for the large segment of the population in the country. Shortages and lack of diversity in clinical specialties in the medical schools can affect the quality of medical education and the current and the future clinical service delivery in the country; hence, the current medical schools and their practice will build the future health human resources of the country. To sustain the quality of medical education, the findings of this study suggest the need for improving the medical education workforce composition and retention through devising various strategies. In addition, different rates of turnover among medical schools might indicate the need for creating uniform and better work environment across the medical schools. Furthermore, attention should be given for health human resources data recording and management throughout the medical schools. And finally, qualitative study is recommended to explore the potential reasons for physician turnover including the observed turnover variations among the medical schools.

## Abbreviations

AAU: Addis Ababa University
AHR: Adjusted hazard ratio
BDU: Bahir Dar University
CPH: Cox proportional hazards
FMOE: Federal Ministry of Education
GNY/OBS: Obstetrics and gynecology
GP: General practitioner
HRH: Human Resources for Health
HR: Human resources
HRIS: Human resource information system
HU: Haromaya University
HwU: Hawassa University
IRB: Institutional review board
JU: Jimma University
MEPI: Medical Education Initiative Partnership
MU: Mekelle University
UOG: University of Gondar
